# Differences between Two Groups of Burmese Vipers (Viperidae: *Azemiops*) in the Proteomic Profiles, Immunoreactivity and Biochemical Functions of Their Venoms

**DOI:** 10.3390/toxins14080572

**Published:** 2022-08-22

**Authors:** Si-Rui Zheng, Yan Sun, Hong-Yan Zhao, Lin Wen, Xiang Ji, Jian-Fang Gao

**Affiliations:** 1 Hangzhou Key Laboratory for Animal Adaptation and Evolution, College of Life and Environmental Sciences, Hangzhou Normal University, Hangzhou 311121, China; 2 Zhejiang Provincial Key Laboratory for Water Environment and Marine Biological Resources Protection, College of Life and Environmental Sciences, Wenzhou University, Wenzhou 325035, China

**Keywords:** venom, *Azemiops*, taxonomy, proteome, biochemical activity

## Abstract

Two recently revised *Azemiops* snakes with apparent differences in their external appearances and skeletal morphologies but unclear genetic boundaries have been proposed. Some researchers have refrained from using the newly proposed taxonomy because these two “species” might be two clades corresponding to different geographical populations of *Azemiops feae*. To improve the understanding of the kinship of these two Burmese viper groups, more of their characteristics should be explored in depth. We performed a comparative analysis of the proteomic profiles and biochemical activities of snake venoms from these two groups (Sichuan *A. feae* and Zhejiang *A. feae*) and evaluated the immunorecognition capacity of commercial antivenoms toward them. Eight protein families were identified in venoms from these two groups, while phospholipase B was only detected in venom from Sichuan *A. feae*. These protein families displayed varying degrees of differences in relative abundance between venoms, and phospholipase A_2_ (Sichuan *A. feae*: 57.15%; Zhejiang *A. feae*: 65.94%) was the predominated component. *Gloydius brevicaudus* antivenom exhibited the strongest capacity to immunologically recognize these two venoms, but this was mainly limited to components with high molecular masses, some of which differed between venoms. Additionally, Zhejiang *A. feae* venom was more toxic than Sichuan *A. feae* venom, and the venoms expressed remarkable differences in enzymatic activities, probably resulting from the variation in the relative abundance of specific protein families. Our findings unveil differences between the two Burmese viper groups in terms of proteomic profiles, immunoreactivity, and the biochemical functions of their venoms. This information will facilitate the management of snakebites caused by these snakes.

## 1. Introduction

Snake venom is an innovative arsenal comprising proteins and peptides. It is considered to help snakes to immobilize, kill, and digest their prey, thus allowing advanced snakes to switch from a mechanical to a chemical means of predation and defence [1]. As a complex mixture, snake venom might possess various toxicological and enzymatic activities that are related to the complicated local and systemic symptoms experienced by victims bitten by venomous snakes [2]. Accordingly, snake venom could potentially serve as a resource library for exploring leading compounds in novel drug design, as well as for the preparation and improvement of antivenoms. Moreover, elucidating the profiles of snake venom could aid in the prediction of toxicological profiles and clinical manifestations of envenoming and facilitate the diagnosis and treatment of snakebites.

On the other hand, the complexity of snake venom is widely considered to be due to adaptations as a result of the diverse dietary sources adopted during the radiation of snakes. Except for a few components that are inferred to be modified products of existing salivary proteins, most snake venom components have evolved from proteins with normal physiological functions through gene recruitment [3,4,5]. Thus, as a special phenotype, venom may retain valuable information about the evolutionary history of venomous snakes, and the proteomic profiles of venom might help with the recognition of differences among related species with taxonomic controversy and the exploration of adaptive evolution trends in the venom compositional pattern along the phylogenetic clues [1,6,7,8,9]. For example, two closely related *Atropoides* taxa from Central America can easily be confused in specimen collection because of their relatively similar external appearances; however, they can be clearly distinguished by the high degree of divergence (at least 80%) in their venom composition by venomic analysis [10]. A further example is the case of three pit vipers in the *Porthidium* group from Costa Rica. Their identification has been controversial, and several changes in their taxonomic relationship and designation have occurred over time [11]. In the latest revision, the phylogenetic divergence among the snakes within this group was clarified by an in-depth molecular analysis, a process that is closely related to the interspecific differences in the venomic profiles of these species [11,12]. As neither basal nor derived taxa within the clades show one venom type (I or II) preferentially, the venom type in *Crotalus* seems to be unrelated to phylogeny [13]. Nevertheless, a paedomorphic trend as to the gain of neurotoxicity-associated lethal venom activity against mammals has been discovered in the *Crotalus durissus* complex and *Crotalus simus*, which is then inferred to emerge during the invasion and speciation of these snakes in South America [14]. Similarly, the dichotomy of venom phenotypes regarding the relative abundance of phospholipase A_2_ (PLA_2_) and three-finger toxin (3-FTx) has been elucidated in *Micrurus* coral snakes and is considered to be a phylogeography-associated trend in relevant species along a North-South axis in the Americas [7].

After the Burmese viper *Azemiops feae* was found and designated in 1888 by Boulenger, it was considered the only member of the monotypic genus *Azemiops* in the family Viperidae for a long period of time. Later, it was assigned to the Azemiopinae subfamily in 1971 by Liem et al. [15,16]. Apparent divergences in external appearances and skeletal morphologies between two groups from the genus *Azemiops* were recently proposed, and this genus has subsequently been revised to comprise two species, the black-headed Burmese viper *A. feae* and white-headed Burmese viper *Azemiops kharini*, with the head color being the designation symbol [15,17]. The distribution regions of these two species are roughly divided by a geographical line running through Hanoi in Vietnam and Kunming and Panzhihua in China, the east of which is mainly occupied by *A. kharini* and the west of which is occupied by *A. feae* [15]. The newly revised taxonomy is being accepted [18]. However, another more recent investigation declared that these two proposed Burmese vipers could not be recovered using monophyly, as their genetic boundary is still not clear based on a unified multilocus molecular marker. Thus, they might probably be two clades corresponding to different geographical populations [19]. Thus, some researchers have refrained from using this newly proposed taxonomy [20,21,22]. Here, for consistency with several recent publications [20,21,22,23], we continue to refer to the snakes from the genus *Azemiops* as *A. feae* and discriminate between snakes with different head characteristics as black-headed Burmese vipers and white-headed Burmese vipers.

**Figure 1:**
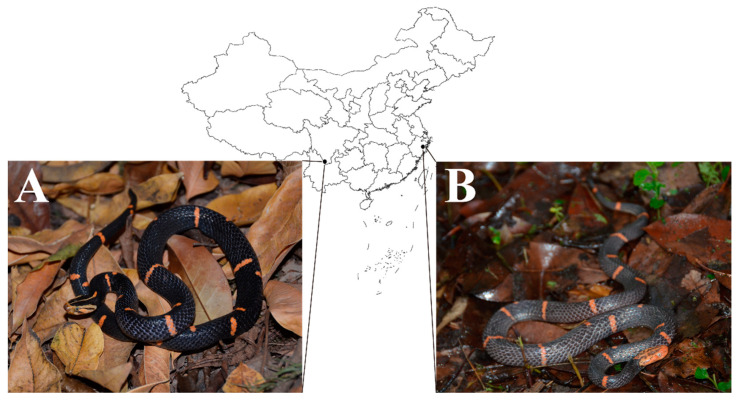
Sampling localities of the two Burmese vipers investigated in this study. (**A**) Sichuan *Azemiops feae* has a black head surface with a thin, yellow strip down the middle. (**B**) Zhejiang *A. feae* has a light head surface divided by two symmetrical dark stripes. The animal images were photographed by Jian-Fang Gao.

Given the potential value of proteomic profiles and the biochemical functions of snake venom in the abovementioned taxonomy, profiling the relevant profiles of black-headed Burmese viper and white-headed Burmese viper might provide some implications for the exploration of their kinship. Although a recent -omics investigation described the detailed venomic profile of Vietnamese *A. feae* at the molecular and protein levels [21], no detailed information is available to explore the potential differences between the venoms from these two Burmese viper groups. Therefore, a conventional strategy that combined RP-HPLC, SDS-PAGE, MALDI-TOF-MS/MS, and nESI-MS/MS was employed in the present study to elucidate and compare the Sichuan *A. feae* (black head) and Zhejiang *A. feae* (white head) (Figure 1) venom proteomes, as well as their toxicological and enzymatic activities. In addition, a cross-reaction between these two venoms and four commercial monovalent antivenoms was also conducted. Our findings might have implications for not only evaluating the kinship of these two Burmese viper groups but also for determining the efficiency of commercial antivenom for use in the clinical treatment of the envenomation caused by them.

## 2. Results and Discussion

### 2.1. Proteomic Profiles of Venoms from Two A. feae Groups

Over the past two decades, a conventional strategy that combined RP-HPLC, SDS-PAGE, and MS identification has been widely employed to explore the variations in the proteomic profiles of snake venoms both between and within species [24,25,26]. Some investigations have also performed N-terminal sequencing, MS-based apparent molecular mass determination, and protein similarity coefficient calculation to estimate the differences in toxin proteins from different snake venoms [1,10]. The current study used a conventional strategy to reveal the differences between the proteomic profiles of pooled venoms from two Burmese viper groups.

**Figure 2:**
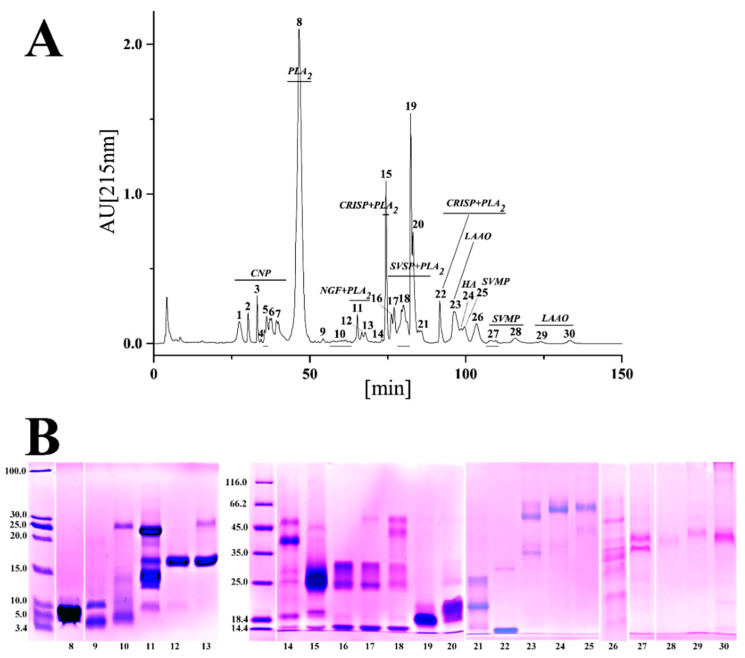
The venom proteomic profile of Sichuan *A. feae*. (**A**) Elution profile of venom proteins in RP-HPLC. The venom components were separated by a C18 column as described in the Materials and Methods section. (**B**) Electrophoretic profile of eluted fractions under reduced conditions. The protein bands were excised, tryptic digested, and analyzed by MALDI-TOF-MS/MS and nESI-MS/MS before being assigned to known protein families. CNP: C-type natriuretic peptide; CRISP, cysteine-rich secretory protein; HA: hyaluronidase; LAAO: l-amino acid oxidase; NGF: nerve growth factor; PLA_2_, phospholipase A_2_; PLB: phospholipase B; SVMP: snake venom metalloproteinase; SVSP: snake venom serine proteinase.

**Figure 3:**
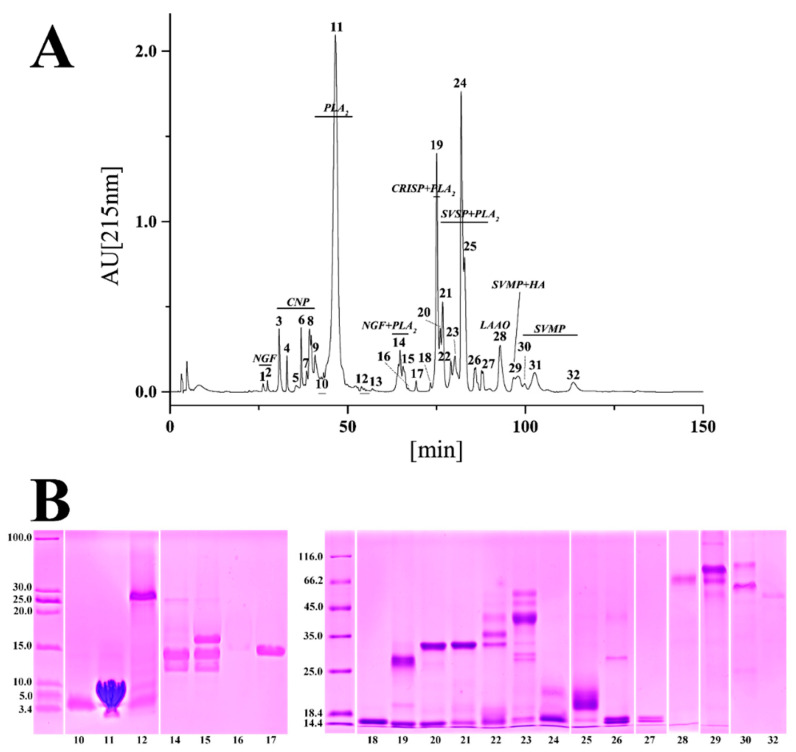
The venom proteomic profile of Zhejiang *A. feae*. (**A**) Elution profile of venom protein in RP-HPLC. The venom components were separated by a C18 column as described in the Materials and Methods section. (**B**) Electrophoretic profile of eluted fractions under reduced conditions. The protein bands were excised, tryptic digested, and analyzed by MALDI-TOF-MS/MS and nESI-MS/MS before being assigned to known protein families.

**Figure 4:**
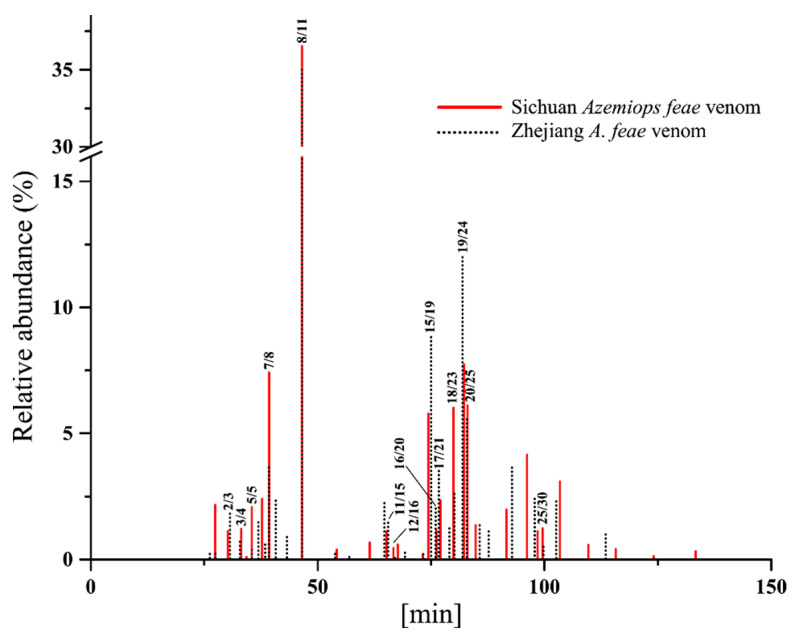
Comparison of simplified elution profiles in RP-HPLC between venoms from two *A. feae* groups. *x*-axis, retention time; *y*-axis, relative abundance of each chromatographical fraction in the total venom. The numbers (Sichuan *A. feae*/Zhejiang *A. feae*) at the end of the lines correspond to the fractions in the chromatography shown in Figure 2 and Figure 3 and indicate similar retention times and protein families for both venoms.

Specifically, a total of 30 chromatographic fractions were resolved in Sichuan *A. feae* venom by RP-HPLC, and 66 protein bands from 23 fractions were separated by SDS-PAGE and identified by MALDI-TOF-MS/MS and nESI-MS/MS analysis. Seven chromatographic fractions with low retention times were directly identified by nESI-MS/MS analysis (Figure 2 and Supplemental Table S1). Fractions 1–7 purely comprised C-type natriuretic peptide (CNP) and accounted for 16.47% of all venom components. Fraction 8 was identified as PLA_2_ with a high abundance (36.52%), while most of the other fractions with relatively high levels of abundance comprised more than one toxin family (Figure 2A and Supplemental Table S1). A total of 32 chromatographic fractions were resolved from Zhejiang *A. feae* venom by RP-HPLC, and 47 protein bands were identified from 21 fractions separated by SDS-PAGE and identified by MALDI-TOF-MS/MS and nESI-MS/MS analysis. Ten chromatographic fractions with low retention times and one with a high retention time were directly identified by nESI-MS/MS analysis (Figure 3 and Supplemental Table S2). Two forerunner fractions were identified as nerve growth factor (NGF) with low abundance (0.46%). The following six fractions (3–8) only comprised CNP (8.64%), and fraction 11 was highly abundant in PLA_2_ (35.00%). Most of the fractions with relatively high abundance comprised more than one toxin family (Figure 3B and Supplemental Table S2). Comparatively speaking, nearly half of the chromatographic fractions (18) expressed similar retention times for both venoms and accounted for 84.21% and 81.20% of the total venom fractions in Sichuan *A. feae* and Zhejiang *A. feae*, respectively (Figure 4 and Supplemental Table S3). Among them, 14 fractions accounting for 74.20% and 72.55% of the total venom fractions comprised similar protein families in both venoms, for example, PLA_2_ in fractions 8/11 (Sichuan *A. feae*/Zhejiang *A. feae* venom), cysteine-rich secretory protein (CRISP) and PLA_2_ in fractions 15/19, and SVSP and PLA_2_ in fractions 16–20/20–25, although some of these fractions differed in terms of the relative abundance of specific protein families (Figure 4, Supplemental Tables S1–S3).

**Figure 5:**
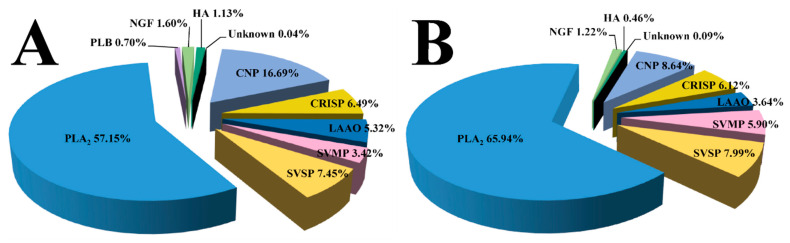
Relative abundance of venom toxin families in Sichuan *A. feae* (**A**) and Zhejiang *A. feae* (**B**). Unknown: unidentified components. The details of the identified venom proteins are listed in Supplemental Tables S1 and S2.

In agreement with the viewpoint that the venom proteomes mainly comprise components belonging to a few protein families [10,24,25,27,28], the proteins from all fractions in Sichuan *A. feae* and Zhejiang *A. feae* venoms could be clustered into nine and eight different toxin families, respectively, including CNP, CRISP, l-amino acid oxidase (LAAO), snake venom metalloproteinase (SVMP), snake venom serine proteinase (SVSP), PLA_2_, phospholipase B (PLB), NGF, and hyaluronidase (HA) (Figure 5). Of note, eight protein families were found to be shared between both groups, while PLB was only detected in Sichuan *A. feae* venom. Similar to the conclusion that *A. feae* venom is primarily composed of PLA_2_ at the mRNA level [22], PLA_2_ was identified as the predominate protein family in both venoms with a relative abundance exceeding 57% higher than that expressed in the venom proteomes of most snakes [29]. Five protein families in each snake venom proteome expressed a relative abundance of more than 5%, and several protein families expressed high divergence in relative abundance between the two venoms, for example, the relative abundance of HA and CNP in Sichuan *A. feae* venom were found to be nearly 2.5- and 1.9-fold of those in Zhejiang *A. feae* venom, respectively. In contrast, an investigation based on the label-free quantitative proteomic method revealed that venom from a Vietnamese *A. feae* specimen is mainly composed of 13 protein families, of which SVSP (44.8%) and PLA_2_ (25.8%) are the predominant components expressed, and the CNP is expressed with a relatively low abundance of 3.0% [21]. The divergence in venom composition between the Vietnamese *A. feae* and our *A. feae* might be attributed to the different strategies employed for proteomic identification and relative abundance quantification, as well as the different geographical origins of the venom samples.

Moreover, sequence alignment has found a relatively low amino acid identity of 84% in plasminogen-activator-like serine proteinase homologues between Chinese *A. feae* (most likely *A. kharini* [23]) and Vietnamese *A. feae*, which is very close to those between Chinese *A. feae* and other pitvipers [20]. Remarkably, the proteins from the two venoms investigated in the current study were found to be best matched to 34 and 27 entries in the database, respectively, of which only 13 were shared between both, accounting for 38.2% and 48.1% of the total identified proteins and 84.25% and 85.97% of the relative abundance (Supplemental Tables S2 and S3). Using a protein similarity coefficient (PSC) [1], we estimated that these two Burmese viper groups share approximately 43% of their venom proteomes, higher than the values estimated within the genera *Atropoides* (14–16%) [10] and *Bitis* (7–28%) [1], but lower than that within the genus *Bothrops* (65–70%) [30]. This might imply a moderate level of interspecific variation in venom proteomes between Sichuan *A. feae* and Zhejiang *A. feae*. However, the criteria applied in the current study to determine whether members of any pair of toxin proteins are the same or highly similar were not as strict as those used in previous studies [1,10]. Therefore, it was relatively rough to illustrate the difference in venom composition by PSC between the Burmese viper groups. In view of the fact that the composition of snake venom may also be affected by various factors, such as the individual specimen, gender, and ontogeny [31,32,33,34], the venom compositions of these two Burmese viper groups should be further clarified at these levels.

### 2.2. Immunorecognition by Commercial Antivenoms

**Figure 6:**
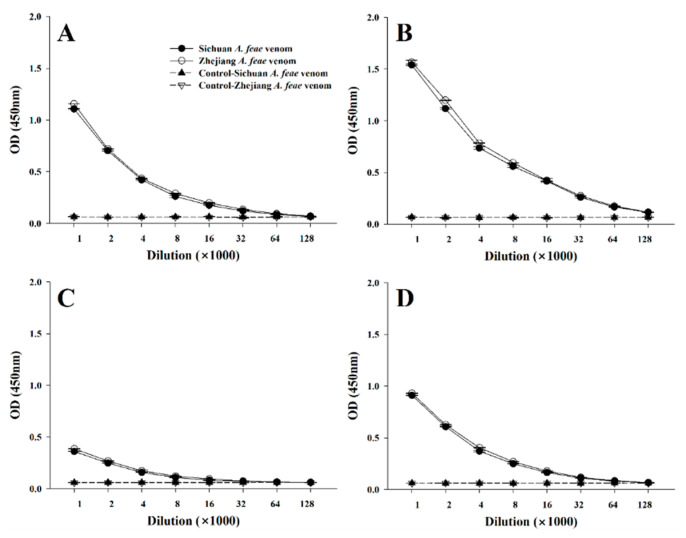
Cross-reaction between two Burmese viper venoms and four commercial antivenoms evaluated by ELISA. (**A**–**D**): commercial monovalent *Deinagkistrodon acutus*, *Gloydius brevicaudus*, *Bungarus multicinctus* and *Naja atra* antivenoms, respectively. The explanation for different lines in (**B**–**D**) is the same as that in (**A**). Data are expressed as the mean value ± SD (*n* = 3).

Accidental envenomation caused by Burmese vipers is relatively rare, and it is not paid enough attention in the clinic [35,36]. Victims bitten by Burmese vipers always suffer from some slight local symptoms, such as pain, swelling, minor bleeding, and ecchymoma around the wound, as well as several marked systemic symptoms, including dizziness and nausea, vomiting, ptosis, blurred vision, and slurred speech [37,38,39]. Thus far, no commercial species-specific antivenom against Burmese viper venoms is available to treat the relevant envenomation. In mainland China, one heterologous commercial antivenom or a combination of two is often employed for the clinical treatment of envenomation caused by *Azemiops* snakes [37,40,41], of which the *G. brevicaudus* antivenom has been found to display the best therapeutic efficacy [37]. Here, we verified by an ELISA test that the *G. brevicaudus* antivenom possesses the strongest capacity to immunorecognize the venoms from these two Burmese viper groups. Briefly, the Sichuan *A. feae* and Zhejiang *A. feae* venoms were immunorecognized by four monovalent commercial antivenoms that prevail in mainland China (Figure 6). Along with the rising of the antivenom concentration, the cross-reaction between venoms and antivenoms gradually increased, with that of Sichuan *A. feae* venom being slightly weaker than that of Zhejiang *A. feae* venom. Moreover, the cross-reaction induced by Viperidae antivenoms was stronger than that of Elapidae antivenoms, and that of *G. brevicaudus* antivenom was the strongest in all ELISA tests. It was found that, at a dilution of 1:1000, the efficiency of *G. brevicaudus* antivenom in recognizing both two venoms (Sichuan *A. feae*/Zhejiang *A. feae*) was nearly 1.4/1.4-fold, 4.3/4.1-fold, and 1.7/1.7-fold higher than that of the *D. acutus*, *B. multicinctus* and *N. atra* antivenoms, respectively (Figure 6). Accordingly, the employment of *G. brevicaudus* antivenom should be preferentially encouraged for the treatment of envenomation caused by *Azemiops* snakes when no species-specific antivenom is available in mainland China.

**Figure 7:**
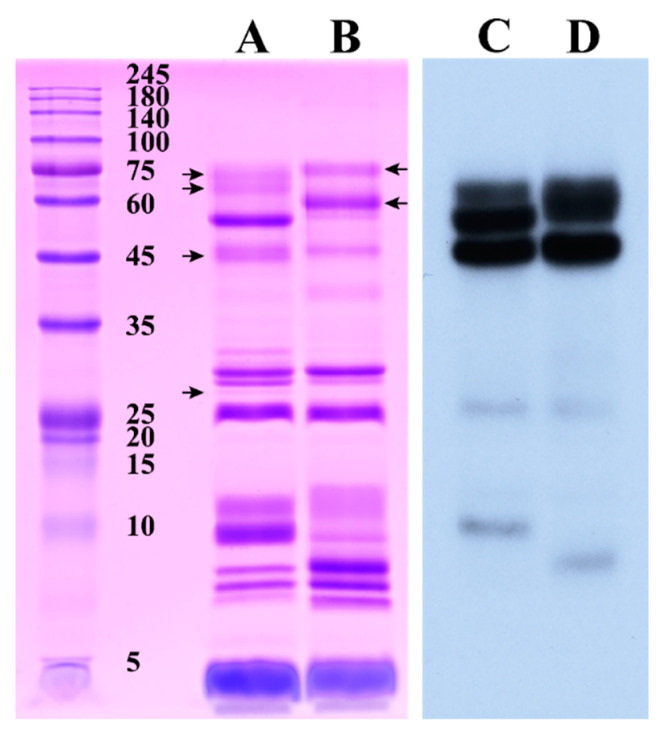
Cross-reaction between two Burmese viper venoms and commercial *G. brevicaudus* antivenom assessed by Western blotting. Left panel, SDS-PAGE profiles of venom protein (identified proteins were listed in Supplemental Tables S3 and S4); right panel, cross-reaction profiles of Western blotting; (**A**,**C**), Sichuan *A. feae* venom; (**B**,**D**), Zhejiang *A. feae* venom. The protein bands specifically expressed in both venoms are indicated by arrows, which on the left of lane A indicate the protein bands with molecular mass of ~27, 45, 66 and 73 kDa from bottom to top, and those on the right of lane B indicate ~59 and 75 kDa.

Because the strength of the cross-reaction between venom and antivenom in the ELISA test was only roughly indicated by the OD values, the potential variation in immunoreactivity between these two venoms investigated in the present study might have been neglected if there were similar OD values. Consequently, we conducted an in-depth analysis of the immunorecognition capacity of *G. brevicaudus* antivenom toward these two venoms by Western blotting, between which the apparent differences in protein bands and the relevant protein families were notably unveiled. The electrophoresis analysis resolved three composition areas with protein bands of ~6.7–11, ~25.5–35, and ~39–75 kDa and one band with a low molecular mass (~4.3 kDa) in both venoms (Figure 7). Except for a small number of protein bands (~27, 45, 66, and 73 kDa in Sichuan *A. feae* venom; ~59 and 75 kDa in Zhejiang *A. feae* venom; indicated by arrows in Figure 7), most of the protein bands were expressed in both venoms. However, almost all protein bands common to both venoms showed significant differences in terms of their relative abundance. Only a small number of venom components were immunorecognized by commercial *G. brevicaudus* antivenom, and the cross-reactivity was even negatively correlated with the abundance of these venom components (Figure 7). Specifically, the strong cross-reaction was mainly located in the protein bands with high molecular masses (~45/47, 54, and 66 kDa in Sichuan *A. feae* venom; ~47 and 59 kDa in Zhejiang *A. feae* venom), and weak cross-reactions were detected in two protein bands with relatively low molecular masses (~9.5 and 25.5 kDa; ~8 and 25.5 kDa) in each venom. It was further revealed by mass spectrometry analysis that these protein bands cross-reacted with the commercial *G. brevicaudus* antivenom could be assigned to more than one protein family, where LAAO, SVMP, SVSP, CRISP, and PLA_2_ accounted for high percentages according to the peptide intensity (Supplemental Tables S4 and S5). Several protein bands with relatively high abundance in both venoms presented no cross-reactivity with the commercial *G. brevicaudus* antivenom, such as those with molecular masses of ~4.3, 7.3 and 28 kDa, which were predominated by CNP, PLA_2_ and SVSP, respectively (Figure 7, Supplemental Tables S4 and S5). Nevertheless, it could not be clarified whether there are more potential differences in immunoreactivity between these two venoms, and this could be further explored through cross-reactions with the other three commercial antivenoms by Western blotting or even an antivenomic strategy.

### 2.3. Toxicological and Enzymatic Activities of Both Venoms

**Figure 8:**
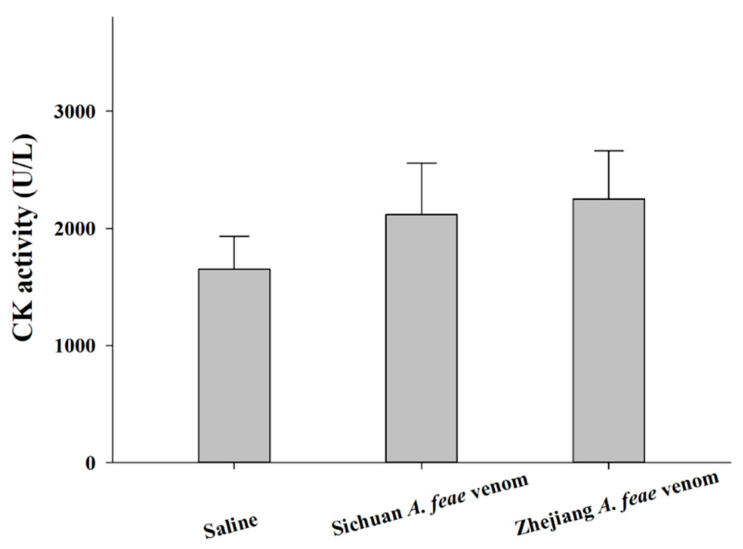
Myotoxicity of Sichuan *A. feae* and Zhejiang *A. feae* venoms in mice. Data are expressed as the mean value ± SD (*n* = 3).

Considering that the clinical symptoms of snakebite might be influenced by venom composition-related biochemical functions, the current study explored the potential variations in toxicological and enzymatic activities between venoms from Sichuan *A. feae* and Zhejiang *A. feae* to promote the understanding of clinical symptoms of snakebites from these species. Similar to the observation of relatively high toxicity (i.v. LD_50_ of 0.52 mg/kg, [42]) found in a previous investigation, the venoms of both Burmese viper groups also showed strong levels of toxicity in mice, with the Sichuan *A. feae* venom (i.p. LD_50_ of 0.56 μg/g) being weaker than the Zhejiang *A. feae* venom (i.p. 0.40 μg/g) (Table 1). The systemic envenoming manifestation of mice injected with Zhejiang *A. feae* venom was much stronger than that of mice injected with Sichuan *A. feae* venom, with manifestations including apparent respiratory depression, convulsion and bradykinesia. Consistent with the observation of slight or even no hemorrhagic activity or myotoxicity in experimental mice injected with *A. feae* venom [40,41], the statistical results of the current study indicate no difference in myotoxicity between venoms and saline (Sichuan *A. feae*: *t* = 1.54, *df* = 4, *p* = 0.20; Zhejiang *A. feae*: *t* = 2.09, *df* = 4, *p* = 0.10). However, it seems that the venoms (Sichuan *A. feae*: 2116.4 ± 254.1 U/L; Zhejiang *A. feae*: 2250.4 ± 236.5 U/L) can cause apparent myotoxicity in mice when compared with saline treatment (1652.0 ± 161.7 U/L) (Figure 8). Moreover, the gastrocnemius muscles of mice injected with venom showed no local symptoms, such as intramuscular hemorrhage. It was also found that neither venom induced hemorrhagic symptoms in the dorsal skin (Table 1) but could cause clear subcutaneous telangiectasia around the injection site. This is consistent with the previous viewpoint [42] that both *Azemiops* venoms appear to be “atypical” viperid venoms.

**Figure 9:**
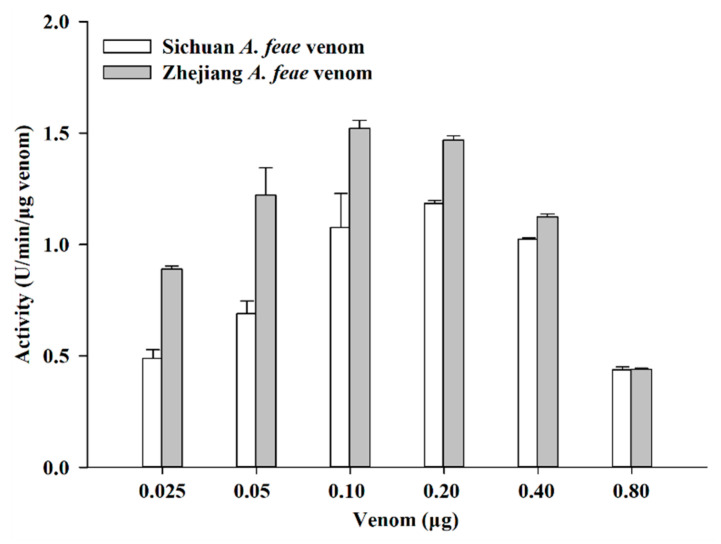
Phospholipase A_2_ activity of Sichuan *A. feae* and Zhejiang *A. feae* venoms determined using soybean lecithin. Data are expressed as the mean value ± SD (*n* = 3). It can be inferred that the substrate was greatly degraded by venoms at relatively high doses, and thus, the activity values of venoms appear to decrease significantly at 0.4 and 0.8 μg.

Variations in venom composition and biochemical properties are always related to differences in the presence or absence and the relative amounts of the components. Here, Zhejiang *A. feae* venom was found to be about 1.3- and 2.4-fold more active in terms of proteolytic and esterolytic activity than Sichuan *A. feae* venom, respectively (all *p* < 0.0001, Table 1). This may be attributed to the relatively higher abundances of SVMP and SVSP in Zhejiang *A. feae* venom. Except for hemorrhagic activity, SVMP is considered to be responsible for a number of other activities, such as prothrombin activating, blood coagulation factor X activating, apoptotic activity, and so on [43]. However, no apparent hemorrhagic activity could be detected in the present study; thus, SVMPs in these two Burmese viper venoms might express other activities mentioned above. SVSP mainly affects the hemostatic system of the victim through a coagulation cascade [44], and three thrombin-like or kallikrein-like enzymes have been predicted by bioinformatic analyses in a recent study [20]. However, it has been found that the *A. feae* venom can not affect clotting [45]. Thus, a detailed investigation of the anticoagulant activity of both Burmese viper venoms should be conducted in the future. In contrast, the Sichuan *A. feae* venom contained a higher abundance of LAAO and thus expressed nearly 1.3-fold more active in LAAO activity than Zhejiang *A. feae* venom (*p* < 0.0001, Table 1). The venoms of both Burmese viper groups showed apparent PLA_2_ activity in degrading the soybean lecithin, which gradually enhanced at first and then weakened with the increase in venom doses ranging from 0.025 to 0.8 μg (Figure 9). Moreover, Sichuan *A. feae* venom can degrade the substrate more slowly than Zhejiang *A. feae* venom at doses of 0.025 to 0.2 μg (all *p* < 0.01). On the other hand, a reaction plateau was reached very quickly at higher venom doses (0.4 and 0.8 μg), which might have been due to substrate depletion during the measurement. Accordingly, it would have been difficult to evaluate the initial velocities, and a comparison of the PLA_2_ activity of venoms at these two doses was not conducted in this study. In total, the Sichuan *A. feae* venom expressed relatively weaker PLA_2_ activity than the Zhejiang *A. feae* venom, which was mainly related to the difference in the relative abundance of PLA_2_ between venoms. The PLA_2_s annotated from *A. feae* venom gland transcripts have been deduced to contain myotoxic, edematous and antimicrobial (Af-N49b), antiplatelet (Af-N49a, Af-N49a1 and Af-E6) activities, whereas the Af-N49b has not been detected at the protein level [23]. Here, no myotoxicity in mice might be due to the lack of Af-N49b-like components in venoms from these two Burmese viper groups. The blast results indicated most of the PLA_2_s in the present study were highly similar to Af-N49a1 and Af-E6, especially in Sichuan *A. feae* venom (accession A0A0D3N944 and A0A0D3N8V5; Supplemental Tables S1 and S2); thus the venom might express high antiplatelet activity. Although more abundant hyaluronidase was detected in Sichuan *A. feae* venom than in Zhejiang *A. feae* venom, the venoms showed no difference in the ability to degrade hyaluronic acid (*t* = 0.06, *df* = 4, *p* = 0.96).

## 3. Conclusions

We used a conventional proteomic strategy to explore the venom compositions of Sichuan *A. feae* and Zhejiang *A. feae* from mainland China and found that these two venoms contained similar general classes of proteins but differed in their relative abundances of specific protein families. Both venoms contained a high abundance of PLA_2_ but only shared approximately 43% of their venom proteomes. This might imply that there is a moderate level of interspecific variation in venom proteomes between these two Burmese viper groups. Although four commercial antivenoms from mainland China were able to recognize the venoms from these two Burmese viper groups, as determined by an ELISA test, *G. brevicaudus* antivenom was verified to possess the strongest immunorecognition capacity. Moreover, Sichuan *A. feae* venom showed a slightly stronger effect than Zhejiang *A. feae* venom in the cross-reaction with these antivenoms. Notably, the protein bands and the relevant protein families of both venoms immunorecognized by *G. brevicaudus* antivenom did not show consistent results in the Western blotting test. In clinical management, it should be preferentially encouraged to employ *G. brevicaudus* antivenom to treat the envenomation caused by *Azemiops* snakes when no species-specific antivenom is available in mainland China. Apparent differences in the biochemical properties of venoms were also detected in these two groups and should be attributed to the differences in their venom compositions. As a whole, it is worth trying to apply the venom proteomic profiles, immunoreactivity, and biochemical functions as assistant tools for investigating the kinship of closely related venomous snakes.

## 4. Materials and Methods

### 4.1. Animals, Venoms, Antivenom, and Ethics

Five and four *A. feae* of both sexes were collected from Panzhihua, Sichuan and Taizhou, Zhejiang, China, respectively, and transferred to the Herpetological Research Center at Hangzhou Normal University. The snakes were maintained in reptile pet terrariums (60 × 45 × 45 cm, Reptizoo) and provided with food and clean water ad libitum. The venom of each snake was extracted using a 100 μL plastic pipette, centrifuged at 10,000× *g* 4 °C to remove impurities, lyophilized, and weighed. Subsequently, the lyophilized venom from the same population was pooled in equal parts and stored at −80 °C prior to analyses. Four commercial monovalent *D. acutus*, *G. brevicaudus*, *B. multicinctus*, and *N. atra* antivenoms comprising purified F(ab′)_2_ from hyperimmunized horses were purchased from Shanghai Serum Biological Technology Co., Ltd., Shanghai, China. The antivenoms were lyophilized and stored at −80 °C as soon as they had been received by the laboratory. The collection of all snakes, utilization of the male ICR mice (supplied by Laboratory Animal Center of Hangzhou Normal University) and the experimental procedures complied with the protocols and guidelines approved by the Animal Research Ethics Committee of Hangzhou Normal University (AREC2019109).

### 4.2. Separation of Venom Proteins by RP-HPLC and SDS-PAGE

The lyophilized venom (5 mg) was reconstituted in 0.1% TFA and centrifuged at 10,000× *g* 4 °C for 15 min; then, the supernatant was automatically loaded onto a Kromasil 300 RP-C18 column (250 × 4.6 mm, 5 μm; AkzoNobel, Bohus, Sweden) and separated at a flow rate of 1 mL/min using a Waters E2695 HPLC system (Waters, Milford, MA, USA). The process was automatically run under a linear gradient of mobile phase A (0.1% TFA) and B (100% ACN) according to Gao et al. [24]: isocratically (10% B) for 10 min, 10–15% B for 10 min, 15–45% B for 80 min, and 45–60% B for 50 min. The eluted proteins were monitored at 215 nm. The chromatographical fractions were collected manually and concentrated in a Labconco CentriVap Centrifugal Concentrator (Labconco, Kansas, MO, USA). The protein concentration of each fraction was quantified according to Bradford [46]. Subsequently, the proteins in each fraction were re-dissolved in ddH_2_O, mixed with loading buffer and separated by 12% and 18% SDS-PAGE under reducing conditions. The gels were stained in 0.2% CBB R-250 and scanned using a Umax2100 densitometer (Novax Technologies, Taipei, China).

### 4.3. Tryptic Digestion and MS Identification of Venom Proteins

The protein bands of interest were excised from the gels, rinsed with ddH_2_O, and split into pieces before being thoroughly destained with 0.1 mM NH_4_HCO_3_ in 30% ACN and incubated with 0.1 mM NH_4_HCO_3_ at room temperature for 15 min. After being reduced and alkylated by sequential incubation with 50 mM DTT and 55 mM IAA in 0.1 M NH_4_HCO_3_, the proteins in the gel pieces were digested with trypsin (MS grade, Promega, Madison, WI, USA) for 16 h at 37 °C. Then, the peptide mixture in the supernatant was collected, lyophilized, and stored at −80 °C until use. For the MALDI-TOF-MS/MS analysis, 1 μL of peptide mixture re-dissolved in 20% ACN was spotted onto a stainless steel target plate and air-dried, then added to 0.5 μL of 5mg/mL α-cyano-4-hydroxycinnamic acid (ABI) in 0.1% TFA and 50% ACN. After being completely air-dried, the sample mixture was subjected to the 4800 Plus MALDI-TOF/TOF-MS mass spectrometer (AB SCIEX, Framingham, MA, USA) according to the operation manual. For the LC-MS/MS analysis, the peptide mixture was re-dissolved in 0.1% TFA, automatically loaded onto the Ultimate 3000 RSLCnano system, and desalted and enriched using an Acclaim PepMap 100 C18 column (Trap Cartridge; 5 × 0.3 mm, 5 μm; ThermoFisher, Waltham, MA, USA). Subsequently, the mixture was separated using an Acclaim PepMap RSLC 100 C18 column (NanoViper; 75 μm × 15 cm, 2 μm; ThermoFisher, Waltham, MA, USA) in the system with solution A (0.1% FA) and B (20% ACN in 0.1% FA) under the following linear gradient: 4% B for 3 min, 4–50% B for 47 min, 50–99% B for 4 min, and 99% B for 6 min. Peptide eluents were sequentially subjected to a Q Exactive Orbitrap platform (ThermoFisher, Waltham, MA, USA) according to the instruction manual. Since fractions with short retention times could not be effectively detected using SDS-PAGE, they were re-dissolved, digested in solution, and identified directly by LC-MS/MS. The original MS/MS data were interpreted using FlexAnalysis 3.4 (Bruker Daltonics, Brerica, MA, USA) or Xcalibur 2.2 software (ThermoFisher, Waltham, MA, USA), and the sequence similarity was conducted using PEAKS X against the UniProt database with the taxonomy limited to “Serpentes”. The fragment mass tolerances for MALDI-TOF-MS/MS and LC-MS/MS were set at 0.4 Da and 0.1 Da, respectively; carbamidomethyl (C) and oxidation (M) were set as fixed and variable modifications, respectively.

### 4.4. Estimation of Protein Relative Abundance and Protein Similarity Coefficient

The protein relative abundance was calculated according to two previous studies [25,47]. Briefly, the relative abundance of each fraction was estimated based on the integration of peaks in the RP-HPLC conducted by Empower software, and that of each protein band was quantified based on their densitometry in the SDS-PAGE electropherogram. For the fraction with only one protein band in SDS-PAGE, the relative abundance was directly appointed from the integration of peak; for the fraction with various protein bands in SDS-PAGE, the relative abundance of each band was estimated by densitometry using Gel-Pro Analyzer 4.0 software (Media Cybernetics, Rockville, MD, USA). The relative abundances belonging to the same protein family were subsequently accumulated. The similarity of venom proteins between two Burmese viper groups (“a” and “b”) was estimated by the protein similarity coefficient (PSC), which is defined in a previous study [1] and calculated in the following step: PSC_ab_ = (2 × (number of proteins shared between a and b)/(total number of distinct proteins in a + total number of distinct proteins in b)) × 100.

### 4.5. Enzyme-Linked Immunosorbent Assay (ELISA)

Aliquots (100 μL) of venom proteins (2 μg/mL in 0.1M Na_2_CO_3_-NaHCO_3_, pH 9.6) were added to the 96-well microplates and absorbed at 4 °C overnight. The unbound proteins were washed off with PBST (0.05% Tween-20 in 10 mM PBS, pH 7.4), and 150 μL of 2% non-fat milk powder in PBST was added to each well and incubated at 37 °C for 1 h. The plates were rinsed thoroughly three times, and then 100 μL of serially diluted antivenoms (initial concentration 4 μg/μL) in PBST containing 1% BSA was added to each well and incubated at 37 °C for another 1 h. Ordinary horse serum was used as a negative control. The plates were rinsed again, and 100 μL of HRP-labelled anti-horse IgG (1:10,000 dilution, Sigma, Saint Louis, MO, USA) in PBST containing 1% BSA was added to each well and incubated at 37 °C for 1 h. Subsequently, the unbound secondary antibody was thoroughly washed off the plate with PBST. The substrate solution (100 μL, 0.5 mg/mL TMB and 0.006% H_2_O_2_ in 0.15 M citrate buffer system, pH 5.0) was added to each well and incubated at room temperature for 20 min. Finally, 50 μL of 2.5 M sulphuric acid was added to each well to stop the reaction, and the absorbance was recorded at 450 nm using a Varioskan Flash microplate reader (ThermoFisher, Waltham, MA, USA).

### 4.6. Western Blotting

Venom proteins of both snakes were separated by 12% SDS-PAGE under reducing conditions following Laemmli [48]. Subsequently, the proteins on the gels were either stained with CBB R-250 or transferred to a PVDF membrane (0.45 μm, Millipore, Cork, Ireland) by the semi-dry electrophoretic transfer cell (Bio-Rad, Hercules, CA, USA). The membrane was blocked with 5% non-fat milk powder in 20 mM TBST and sequentially incubated with commercial *G. brevicaudus* antivenom (reconstituted in ddH_2_O and diluted by 2% BSA in 1:2000) at 37 °C for 1 h and HRP-labelled antihorse IgG (1:5000 dilution) at 37 °C for another 1 h. The unbound secondary antibody was thoroughly washed off, and the membrane was treated with Pierce ECL WB substrate according to the operation manual before being exposed to X-ray film. The gel and X-ray film were scanned using a UMax2100 densitometer, and the profiles of the bands were analyzed with Gel-Pro Analyzer 4.0 software (Media Cybernetics, Rockville, MD, USA). Additionally, the protein bands that were strongly recognized or those with high abundance but not recognized by the commercial antivenom were excised and identified by LC-MS/MS following the abovementioned procedure.

### 4.7. Toxicological and Enzymatic Activities of Venoms

#### 4.7.1. Lethality

The median lethal doses (LD_50_) of both venoms were assessed by male ICR mice (22–26 g). Groups of four mice were injected by the intraperitoneal route with various doses of crude venom dissolved in 100 μL of physiological saline, as previously described [24], and the control group received the same volume of saline without venom. Deaths were recorded at 24 h post-injection, and the LD_50_ was estimated according to the Spearman–Karber method.

#### 4.7.2. Myotoxicity

The venom (10 μg) in 25 μL of saline was injected into the gastrocnemius muscle of the right hind limb in a group of three male ICR mice (22–26 g), while the controls received the same volume of physiological saline. After 3 h, nearly 0.8 mL of blood was collected from each mouse, and the plasma was obtained after centrifugation at 3000× *g* 4 °C. Then, the creatine kinase (CK) activity was determined using a biochemical kit (Batch No. 20220310; Nanjing Jiancheng Bioengineering Institute, Nanjing, China) according to the instruction manual and defined as U/L.

#### 4.7.3. Hemorrhagic Activity

The hemorrhagic activity was determined according to Gao et al. [24] and Gutiérrez et al. [49]. Briefly, four dose groups (1, 2, 4 and 8 μg) of crude venom in 100 μL saline were injected intradermically into the dorsal skin of three groups of male ICR mice (22–26 g). Controls were only injected with saline at the same volume. After 3 h, the mice were sacrificed with CO_2_, and the hemorrhagic manifestation under the skin was checked. If the hemorrhagic areas could be clearly observed, they were recorded and used to calculate the minimum hemorrhagic dose (MHD), which was expressed as the venom dose that could induce a hemorrhagic area with a diameter of 10 mm.

#### 4.7.4. Proteolytic Activity

We determined the proteolytic activity using the methods of Gao et al. [24] and He et al. [50]. The venom (40 μg) in 100 μL of 20 mM PBS was added to 0.5 mL of 2% casein in 0.2 M Tris–HCl, pH 8.5, and incubated at 37 °C for 2 h. Then, 0.6 mL of 0.44 M TCA was added to the reaction solution and incubated at 37 °C for 30 min. After centrifugation at 12,000× *g* 4 °C for 15 min, 0.8 mL of supernatant was collected and mixed thoroughly with 2.0 mL of 0.4 M Na_2_CO_3_ and 0.4 mL of folin reagent (1:2 dilution), and the absorbance was recorded at 660 nm. l-Tyrosine was used as the standard, and one unit of proteolytic activity was expressed as nmol of l-Tyrosine released/min/mg venom.

#### 4.7.5. Esterolytic Activity

We determined the esterolytic activity using a method modified by Tu et al. [51]. The venom (8 μg) in 2 μL of 20 mM PBS was added to 180 μL of 1 mM BAPNA in 0.1 M Tris-HCl, pH 8.0 in a 96-well micro-plate and incubated at 37 °C for 30 min. Then, 18 μL of 30% acetic acid was added to the reaction system, and the absorbance was read at 405 nm. *p*-nitroaniline was used as the standard, and one unit of esterolytic reactivity was defined as nmol of *p*-nitroaniline released/min/mg crude venom.

#### 4.7.6. l-Amino Acid Oxidase Activity

We assayed the l-amino acid oxidase activity following the method presented by Toyama et al. [52]. The venom (0.8 μg) in 1 μL of 20 mM PBS was added to 90 μL of 50 mM Tris-HCl, pH 8.0 containing 0.25 mM l-Leucine, 2 mM o-phenylenediamine and 0.81 U/mL horseradish peroxidase in a 96 micro-plate, and incubated at 37 °C for 1 h. Then, 50 μL of 2 M H_2_SO_4_ was added to the reaction mixture, and the absorbance was read at 490 nm. H_2_O_2_ was used as the standard, and one unit of l-amino acid oxidase activity was expressed as nmol of H_2_O_2_ degraded/min/mg venom.

#### 4.7.7. Hyaluronidase Activity

We assayed the hyaluronidase activity according to Antunes et al. [31] with some modifications. Venom samples (20 μg) in 5 μL of 20 mM PBS were incubated with 65 μL of substrate solution system (200 mM acetate buffer, pH 6.0, containing 750 μg/mL hyaluronic acid) at 37 °C for 15 min. After, 130 μL of 2% NaOH containing 2.5% cetyltrimethyl ammonium bromide was added to the reaction system. The absorbance was recorded at 400 nm. High purity hyaluronidase was used as the standard. The hyaluronidase activity was defined in terms of National Formulary Units (NFU)/min/mg venom.

#### 4.7.8. Phospholipase A_2_ Activity

We measured the phospholipase A_2_ activity using the method presented by Gao et al. [24] with a slight modification. Six dose groups (0.025, 0.05, 0.1, 0.2, 0.4 and 0.8 μg) of venom in 1 μL of 20 mM PBS in a 96 micro-plate were gently mixed with the substrate solution consisting of 0.1 M NaCl, 10 mM CaCl_2_, 7 mM Triton X-100, 0.35% soybean lecithin, and 98.8 mM phenol red, pH 8.0. The absorbance was read at 558 nm at 28 °C for 2.5 min. One unit of phospholipase A_2_ activity was expressed as a change in absorbance of 0.3 OD value/min/μg venom.

### 4.8. Statistical Analyses

The LD50 was calculated by the Trimmed Spearman-Karber 1.5 program with a 95% confidence interval. Student’s t-test was used to analyze the other toxicological and enzymatic activities based on Statistica 8.0 (StatSoft Inc., Tulsa, OK, USA). Descriptive statistics were defined as the mean values ± standard deviation (SD), with the significance level set at α = 0.05.

## Figures and Tables

**Table 1:** Comparison of the toxicological and enzymatic activities in venoms from two Burmese viper groups.

*Activity*	Sichuan *Azemiops feae* Venom (sAf)	Zhejiang *A. feae* Venom (zAf)	Statistical Results
*Lethality* (LD_50_) ^a^			
ICR mice (μg/g)	0.56 (0.47–0.68)	0.40 (0.34–0.47)	sAf < zAf
*Hemorrhage* (MHD) ^b^			
ICR mice (μg/g)	None	None	-
*Proteolytic activity* ^b^			
Casein (nM/min/mg)	16.0 ± 0.3	20.7 ± 0.1	*p* < 0.0001, sAf < zAf
*Esterolytic activity* ^b^			
BAPNA (nM/min/mg)	17.5 ± 0.8	42.8 ± 1.1	*p* < 0.0001, sAf < zAf
*l-amino acid oxidase activity* ^b^			
l-Leu (nM/min/mg)	370.1 ± 5.0	295.4 ± 6.0	*p* < 0.0001, sAf > zAf
*Hyaluronidase activity*			
Hyaluronic acid (NFU/min/mg)	9.3 ± 0.3	9.3 ± 0.4	*p* = 0.96, sAf = zAf

^a^ LD_50_: dose that induces death in 50% of mice. Confidence limits (95%) are listed in parentheses. ^b^ Data are expressed as the mean value ± SD (*n* = 3).

## Data Availability

The data presented in this study are available in this article and Supplementary Materials.

## Supplementary Materials

The following supporting information can be downloaded from: https://www.mdpi.com/article/10.3390/toxins14080572/s1, Table S1: Assignment of the RP-HPLC fractions from Sichuan *Azemiops feae* venom to protein families by MALDI-TOF-MS/MS and nESI-MS/MS of selected peptide ions from in-gel digested protein bands separated by SDS-PAGE. CNP: C-type natriuretic peptide; CRISP, cysteine-rich secretory protein; HA: hyaluronidase; LAAO: l-amino acid oxidase; NGF: nerve growth factor; PLA_2_, phospholipase A_2_; P[LB: phospholipase B; SVMP: snake venom metalloproteinase; SVSP: snake venom serine proteinase; Unknown: unidentified components; Table S2: Assignment of the RP-HPLC fractions from Zhejiang *Azemiops feae* venom to protein families by MALDI-TOF-MS/MS and nESI-MS/MS of selected peptide ions from in-gel digested protein bands separated by SDS-PAGE. CNP: C-type natriuretic peptide; CRISP, cysteine-rich secretory protein; HA: hyaluronidase; LAAO: l-amino acid oxidase; NGF: nerve growth factor; PLA_2_, phospholipase A_2_; SVMP: snake venom metalloproteinase; SVSP: snake venom serine proteinase; Unknown: unidentified components; Table S3: A simplified description of chromatographic and proteomic profiles of venoms from two Burmese viper groups; Table S4: Assignment of the protein bands from Sichuan *Azemiops feae* venom cross-reacted or expressed a high abundance but could not cross-react with *Gloydius brevicaudus* antiserum to protein families by nESI-MS/MS; Table S5: Assignment of the protein bands from Zhejiang *Azemiops feae* venom cross-reacted or expressed a high abundance but could not cross-react with *Gloydius brevicaudus* antiserum to protein families by nESI-MS/MS.
