# Supplementary material for: Differences between Two Groups of Burmese Vipers (Viperidae: Azemiops) in the Proteomic Profiles, Immunoreactivity and Biochemical Functions of Their Venoms
Source: Toxins (Basel). 2022 Aug 22;14(8):572. doi: 10.3390/toxins14080572 (PMC9416478; doi:10.3390/toxins14080572)
Supplement: Supplementary file 1 [file toxins-14-00572-s001.zip › toxins-1852458-supplementary-Table S1 and S2.pdf]

Supplementary Materials: Differences between Two Groups of Burmese Vipers (Viperidae: *Azemiops*) in the Proteomic Profiles, Immunoreactivity and Biochemical Functions of Their Venoms

Si-Rui Zheng, Yan Sun, Hong-Yan Zhao, Lin Wen, Xiang Ji and Jian-Fang Gao

Table S1. Assignment of the RP-HPLC fractions from Sichuan *Azemiops feae* venom to protein families by MALDI-TOF-MS/MS and nESI- MS/MS of selected peptide ions from in-gel digested protein bands separated by SDS-PAGE. CNP: C-type natriuretic peptide; CRISP, cysteine-rich secretory protein; HA: Hyaluronidase; LAAO: L-amino acid oxidase; NGF: nerve growth factor; PLA2, phospholipase A2; PLB:.

phospholipase B; SVMP: snake venom metalloproteinase; SVSP: snake venom serine proteinase; Unknown: unidentified components.

Peak	%	MW (kDa)	Peptide Ion		Score	MS/MS-derived sequence	Protein family/species/accession
			m/z	z			
1	2.16	-	581.0	3	84	AAATPQKLAKGRGAAATS A	CNP; *Azemiops feae*; K4IT20
			610.0	3	71	SDSKAAATPQKLAKGRGAA	
			633.7	3	68	SDSKAAATPQKLAKGRGAA	
			608.8	2	66	ASDSKAAATPQKL	
			556.9	2	50	RLKGLAKKGAA	
			627.8	2	47	SLRPEAASGPAAAG	
			599.4	2	45	PRPPRPRPKP	
			682.4	2	44	KPPHQGPRPPRP	
			458.8	2	43	KPPHQGPR	
			857.5	2	42	PPHQGPRPPRPRPKP	
			357.9	3	42	RGVGGGGSRRL	
			521.3	2	37	HQGPRPPRP	
			367.6	3	36	RPPRPRPKP	
			409.2	2	35	GAAATSARL	
			1041.7	1	34	RLKGLAKKGA	
			614.7	3	33	KPPHQGPRPPRPRPK P	
			760.4	2	32	HQGPRPPRPRPKP	

2	1.12	-	720.1	3	93	SDSKAAATPQKLAKGRGAAATS	CNP; *A. feae*; K4IT20
			371.6	3	52	ARLKGLAKKGAA	
			682.4	2	49	KPPHQGPRPPRP	
			410.6	3	46	AKGRGAAATSARL	
			633.7	3	46	SDSKAAATPQKLAKGRGAAA	
3	1.21	-	430.7	2	64	LPPHPHY	CNP; *A. feae*; K4IT20
4	0.10	-	645.4	2	65	KGRGAAATSARLM	CNP; *A. feae*; K4IT20
			552.8	2	59	RGAAATSARLM	
			680.9	2	48	AKGRGAAATSARL	
			637.8	2	47	MDLRTDGKQWR	

Peak	%	MW (kDa)	Peptide Ion		Score	MS/MS-derived sequence	Protein family/species/accession
			m/*z*	*z*			
5	2.07	-	637.8	2	54	DLRTDGKQWR	CNP; *A. feae*; K4IT20
			509.3	2	39	KPPGVYYPP	
			750.4	2	38	QKWGRMVPPKGES	
			454.3	3	34	AKGRGAAATSARL	
						M	
6	2.40	-	491.9	3	54	LAKGRGAAATSARL	CNP; *A. feae*; K4IT20
			557.3	2	51	MGPAAAGVGDGWR	
			750.4	2	49	QKWGRMVPPKGES	
			509.3	2	41	KPPGVYYPP	
			600.8	2	40	SGPAAAGVGDGWR	
			636.3	2	40	ASGPAAAGVGDGWR	
			671.8	2	39	AASGPAAAGVGDGW	
			460.7	2	25	R	
						KPGVYYPP	
7	7.41	-	575.6	3	63	RPEAASGPAAAGVGDGWR	CNP; *A. feae*; K4IT20
			613.3	3	63	LRPEAASGPAAAGVGDGWR	
			736.4	2	50	EAASGPAAAGVGDGWR	
			552.0	3	47	REELSLRPEAASGPAA	
			748.9	2	41	ELSLRPEAASGPAAA	
			588.3	2	40	GKPVQYLPPHP	
			600.8	2	39	SGPAAAGVGDGWR	
			684.9	2	38	ELSLRPEAASGPA	
			400.6	3	37	AREELSLRPEA	
			509.3	2	36	KPPGVYYPP	
			813.4	2	35	EELSLRPEAASGPAAA	
			473.9	3	35	GDEKPVQYLPPHP	
			594.6	3	35	REELSLRPEAASGPAAAG	
			460.7	2	34	KPPGVYYP	
8	36.52	4.7	388.7	2	67	VTGCDPK	PLA_2; *A. feae*; A0A0D3N8V5
9	0.12	10.0	403.7	2	15	AAAICFR	PLA_2; *A. feae*; A7X4P4
	0.27	4.0	1138.5	1	15	SVDFDSESPR	CRISP; *Crotalus horridus*; ~F2Q6E7
			1905.0	1	78	KPEIQNEIVDLHNSLR	
10	0.18	25.4	634.3	2	67	LYCIDSSPAN	SVMP; *Trimeresurus stejnegeri*; ~Q2LD49
			423.9	3	53	K	
						SAECTDRFQR	
	0.04	13.9	634.3	2	70	LYCIDSSPANK	SVMP; *T. stejnegeri*; ~Q2LD49

Peak	%	MW (kDa)	Peptide Ion		Score	MS/MS-derived sequence	Protein family/species/accession
			m/*z*	*z*			
	0.04	10.5	1535.5	1	35	CCFEHDCCYGK	PLA$_2$; *Bothriechis nigroviridis*; ~C0HJL8
	0.20	4.6	517.8	2	43	GCFGLPLDR	CNP; *A. feae*; K4IT20
	0.20		404.7	2	34	AAAICFR	PLA$_2$; *Protobothrops flavoviridis*; ~S6B451
11	0.44	23.3	647.3	2	42	IDTACVCVISR	NGF; *A. feae*; Q2XXL6
			549.3	2	38	NPNPVPSGCR	
	0.10	16.6	1097.5	1	42	NPNPVPSGCR	NGF; *A. feae*; Q2XXL6
			1293.6	1	80	IDTACVCVISR	
			1413.6	1	11	CRNPNPVPSGCR	
			1825.8	1	57	HWNSYCTTTHTYVR	
	0.52	14.4	403.7	2	29	AAAICFR	PLA$_2$; *A. feae*; A7X4P4
			440.2	2	19	AVCECDK	
			436.7	2	17	NNVIVCR	
	0.04	9.3	-	-	-	-	Unknown
12	0.46	16.4	947.4	1	49	EGNQASWR	NGF; *A. feae*; Q2XXL6
			1097.5	1	80	NPNPVPSGCR	
			1293.6	1	78	IDTACVCVISR	
			1413.6	1	28	CRNPNPVPSGCR	
			1825.8	1	77	HWNSYCTTTHTYVR	
13	0.04	27.5	1369.6	1	60	CQFKTAGELCR	PLA$_2$; *Protobothrops mucrosquamatus*; ~XP_015683146
			1737.8	1	78	DGHPCQNNLGYCFR	
	0.56	16.6	947.4	1	54	EGNQASWR	NGF; *A. feae*; Q2XXL6
			1097.5	1	79	NPNPVPSGCR	
			1293.6	1	81	IDTACVCVISR	
			1413.6	1	32	CRNPNPVPSGCR	
			1825.8	1	25	HWNSYCTTTHTYVR	
14	0.03	50.8	1148.6	1	29	MIPCAAKDVK	SVMP; *Hoplocephalus bungaroides*; ~R4FIL3
	0.11	40.5	1259.6	1	76	YSVGIVQDHNK	SVMP; *Echis coloratus*; ~A0A0A1WCK4
	0.01	29.6	1905.1	1	42	KPEIQNEIVDLHNSLR	CRISP; *A. feae*; F2Q6E3

0.01	26.3	1138.5	1	34	SVDFDSESPR	CRISP; *Deinagkistrodon acutus*; ~F2Q6G1
		1553.7	1	10	MEWYPEAAANAER	
		1905.0	1	22	KPEIQNEIVDLHNSLR	
0.04	19.3	947.4	1	28	EGNQASWR	NGF; *A. feae*; Q2XXL6
		1097.5	1	20	NPNPVPSGCR	

Peak	%	MW (kDa)	Peptide Ion m/z	z	Score	MS/MS-derived sequence	Protein family/species/accession
			1293.6	1	66	IDTACVCVISR	
	0.01	16.6	403.7	2	53	AAAICFR	PLA$_2$; *A. feae*; A7X4P4
15	0.04	48.0	1537.7	1	14	MEWYPEAAANAER	CRISP; *A. feae*; F2Q6E3
			1905.0	1	32	KPEIQNEIVDLHNSLR	
	5.24	26.1	1537.7	1	33	MEWYPEAAANAER	CRISP; *A. feae*; F2Q6E3
			1553.7	1	17	MEWYPEAAANAER	
			1905.1	1	95	KPEIQNEIVDLHNSLR	
	0.36	20.2	1138.5	1	50	SVDFDSESPR	CRISP; *C. horridus*; ~F2Q6E5
			1553.6	1	31	MEWYPEAAANAER	
			1905.0	1	134	KPEIQNEIVDLHNSLR	
	0.15	26.6	1535.5	1	40	CCFEHDCCYGK	PLA$_2$; *B. nigroviridis*; ~C0HJL8
16	0.49	31.3	1304.6	1	57	NFQMQLGVHSK	SVSP; *A. feae*; A0A1Y0DIB6
			1429.7	1	32	VLNDDEQTRDPK	
	0.32	25.3	2929.5	1	138	LNSPVHESTHIAPLSLPSSSPTVGSVCR	SVSP; *A. feae*; A0A1Y0DIC1
	0.01	19.2	2929.5	1	108	LNSPVHESTHIAPLSLPSSSPTVGSVCR	SVSP; *A. feae*; A0A1Y0DIC1
	0.32	16.6	1535.5	1	44	CCFEHDCCYGK	PLA$_2$; *B. nigroviridis*; ~C0HJL8
17	0.08	56.1	494.5	3	82	VVGGDECNINDHR	SVSP; *Trimeresurus purpureomaculatus*; ~P0DJF7
			741.8	2	85	VVGGDECNINDHR	
	0.59	31.6	1288.6	1	42	NFQMQLGVHSK	SVSP; *A. feae*; A0A1Y0DIB6
			1304.6	1	56	NFQMQLGVHSK	
	0.74	25.8	1304.6	1	35	NFQMQLGVHSK	SVSP; *A. feae*; A0A1Y0DIB6
	0.94	16.8	1535.5	1	55	CCFEHDCCYGK	PLA$_2$; *B. nigroviridis*; ~C0HJL8
18	0.88	55.3	1594.7	1	80	TLCAGILQGGTDTCK	SVSP; *A. feae*; A0A1Y0DIB5
	1.11	44.3	1003.6	1	46	ALYPGLLEK	SVSP; *A. feae*; A0A1Y0DIB4
			1095.7	1	57	FLVALYTLR	
			2233.2	1	63	NSAHIAPLSLPSSPPSVGSVCR	
			2480.2	1	20	ETYPDVPHCANINIVDHAVCR	
	0.88	31.4	2929.5	1	148	LNSPVHESTHIAPLSLPSSSPTVGSVCR	SVSP; *A. feae*; A0A1Y0DIC1
	0.83	26.2	2929.5	1	174	LNSPVHESTHIAPLSLPSSSPTVGSVCR	SVSP; *A. feae*; A0A1Y0DIC1
	2.32	16.9	1535.5	1	45	CCFEHDCCYGK	PLA$_2$; *B. nigroviridis*; ~C0HJL8
			2093.8	1	36	DATDRCCFEHDCCYGK	
19	7.74	19.2	1505.6	1	6	CCFVHDCCYGK	PLA$_2$; *A. feae*; A0A0D3N8V5
			1728.8	1	49	EVCECDKAAAICFR	

Peak	%	MW (kDa)	Peptide Ion		Score	MS/MS-derived sequence	Protein family/species/accession
			m/z	z			
20	0.15	27.0	918.5	1	35	VYPGLLEK	SVSP; *A. feae*; A0A1Y0DIC1
	5.97	21.2	998.4	1	34	TDPYPYSR	PLA_2; *A. feae*; A0A0D3N944
			1534.5	1	43	CCFEHNCCYGK	
			2330.0	1	117	IAFDTYTFYGCNCGWEGLR	
21	0.54	27.9	1003.6	1	49	ALYPGLLEK	SVSP; *A. feae*; A0A1Y0DIB4
			1095.7	1	54	FLVALYTLR	
			2233.1	1	145	NSAHIAPLSLPSSPPSVGSVCR	
			2480.1	1	98	ETYPDVPHCANINIVDHAVCR	
	0.66	21.9	1095.7	1	36	FLVALYTLR	SVSP; *A. feae*; A0A1Y0DIB4
			2233.2	1	70	NSAHIAPLSLPSSPPSVGSVCR	
	0.16	17.8	1003.6	1	42	ALYPGLLEK	SVSP; *A. feae*; A0A1Y0DIB4
			2233.2	1	104	NSAHIAPLSLPSSPPSVGSVCR	
			2480.2	1	103	ETYPDVPHCANINIVDHAVCR	
22	0.32	31.8	1138.5	1	38	SVDFDSESPR	CRISP; *C. horridus*; ~F2Q6E5
			1553.7	1	4	<u>M</u>EWYPEAAANAER	
			1905.1	1	50	KPEIQNEIVDLHNSLR	
	1.66	16.8	1022.5	1	58	TLHYIYGR	PLA_2; *A. feae*; A7X4P4
			1488.7	1	95	IGLQFYVDAFCR	
			1533.6	1	73	CCFVHDCCYGR	
			1967.9	1	171	NPVTSYSAYGCYCGVGGR	
23	0.77	69.2	1127.6	1	48	AHGWIDSTIK	LAAO; *Demansia vestigiata*; ~A6MFL0
	2.31	57.6	1138.7	1	17	ITFEPPLPPK	LAAO; *Bothrops pictus*; ~X2L4E2
			1294.8	1	35	RITFEPPLPPK	
			1388.7	1	42	KFWEDDGIHGGK	
	0.27	40.3	1138.6	1	10	ITFEPPLPPK	LAAO; *Lachesis muta*; ~J7H670
			1266.8	1	22	ITFEPPLPPKK	
	0.81	37.0	1137.5	1	30	HDDIFAYEK	LAAO; *B. pictus*; ~X2L4E2
			1266.7	1	17	ITFEPPLPPKK	
			1294.7	1	18	RITFEPPLPPK	
24	1.13	63.7	1852.0	1	140	DSTALFPNIYLETVLR	HA; *Agkistrodon piscivorus*; ~A0A194ART0
			1889.9	1	103	HSDSNAFLHLFPDSFR	
			2018.0	1	92	KHSDSNAFLHLFPDSFR	
25	1.01	65.4	1615.5	1	32	ECEIGECCDQCR	SVMP; *E. coloratusf*; ~E9JG68

Peak	%	MW (kDa)	Peptide Ion		Score	MS/MS-derived sequence	Protein family/species/accession
			m/z	z			
	0.23	48.4	1069.4	3	4	KYPCHYDYSEDPDYGMVDHGTICADGK	SVMP; *Bothrops atrox*; ~A0A1L8D666
26	0.18	98.5	567.3	2	38	YPVKPSEEGK	LAAO; *L. muta*; ~J7H670
			422.7	2	36	VAEELKR	
	0.43	41.6	438.7	2	41	STTDLPSR	LAAO; *Gloydius blomhoffii*; ~Q90W54
			567.3	2	33	YPVKPSEEGK	
	0.09	35.5	438.7	2	34	STTDLPSR	LAAO; *Oxyuranus scutellatus scutellatus*; ~Q4JHE3
	0.80	61.1	594.2	2	51	DSCCDAATCK	SVMP; *Gloydius halysi*; ~Q8AWI5
			634.3	2	43	LYCIDSSPANK	
			529.8	2	35	IACEPQNVK	
			402.7	2	32	SAADVTLK	
	0.47	45.6	535.2	2	45	IANMMADSGK	PLB; *Crotalus adamanteus*; ~F8S101
			527.2	2	43	IANMMADSGK	
			700.8	2	38	DQGKVTDMESMK	
	0.23	37.3	535.2	2	60	IANMMADSGK	PLB; *C. adamanteus*; ~F8S101
	0.25	32.2	756.3	2	63	SGPTCGDCPSACDK	CRISP; *A. feae*; F2Q6E3
			569.8	2	43	SVDFDSESPR	
			507.7	2	42	CNEDHSPR	
	0.44	24.7	555.8	2	52	DNLDTYQNK	PLA_2; *Protobothrops mangshanensis*; ~A0A0H3U271
	0.20	20.3	437.7	2	42	NNVIVCR	PLA_2; *A. feae*; A7X4P4
			472.7	2	35	CPEGQEKP	
27	0.26	44.0	1259.6	1	59	YSVGIVQDHNK	SVMP; *E. coloratus*; ~E9JGA2
	0.32	40.2	1259.6	1	64	YSVGIVQDHNK	SVMP; *E. coloratus*; ~A0A0A1WCK4
28	0.41	42.4	1259.6	1	130	YSVGIVQDHNK	SVMP; *E. coloratus*; ~E9JGA2
29	0.14	47.3	569.3	2	58	HDDIFAYEK	LAAO; *G. halys*; ~Q6STF1
			567.3	2	53	YPVKPSEEGK	
			438.7	2	48	STTDLPSR	
30	0.32	44.7	569.3	2	74	HDDIFAYEK	LAAO; *Vipera ammodytes ammodytes*; ~P0DI84
			630.8	2	46	FWEDDGIHGGK	
			438.7	2	44	STTDLPSR	

Methionine oxidation is underlined.

Table S2. Assignment of the RP-HPLC fractions from Zhejiang *Azemiops feae* venom to protein families by MALDI-TOF-MS/MS and nESI- MS/MS of selected peptide ions from in-gel digested protein bands separated by SDS-PAGE. CNP: C-type natriuretic peptide; CRISP, cysteine-rich secretory protein; HA: Hyaluronidase; LAAO: L-amino acid oxidase; NGF: nerve growth factor; PLA2, phospholipase A2; SVMP: snake venom metalloproteinase; SVSP: snake venom serine proteinase; Unknown: unidentified components.

Peak	%	MW (kDa)	Peptide Ion m/z	Peptide Ion z	Score	MS/MS-derived sequence	Protein family/species/accession
			375.6	3	50	VPPKGESVGVR	
			627.8	2	50	SLRPEAASGPAAAG	
			682.4	2	50	KPPHQGPRPPRP	
			599.4	2	49	PRPPRPRPKP	
			565.3	2	48	DSKAAATPQKL	
			610.0	3	46	SDSKAAATPQKLAKGRGAA	
			563.8	2	45	SLRPEAASGPAA	
			529.3	2	45	GRMVPPKGES	
			492.8	2	42	SLRPEAASGP	
			528.3	2	41	SLRPEAASGPA	
			380.7	2	41	KPPHQGP	
			575.8	2	39	TDDTTALREE	
			557.3	3	38	AATPQKLAKGRGAAATSA	
			367.6	3	37	RPPRPRPKP	
			380.9	3	36	ZGPRPPRPRP	
			458.8	2	36	KPPHQGPR	
			921.5	2	35	KPPHQGPRPPRPRPKP	
			556.6	3	35	ZGPRPPRPRPKPSQS	
			455.9	3	34	ZGPRPPRPRPKP	
			600.8	2	34	DSKAAATPQKLA	
			760.4	2	34	HQGPRPPRPRPKP	
4	0.71	-	720.1	3	90	SDSKAAATPQKLAKGRGAAATSA	CNP; *A. feae*; K4J3K2
			627.8	2	51	SLRPEAASGPAAAG	
			682.4	2	49	KPPHQGPRPPRP	
			771.4	1	48	ZKPPGVY	
			410.6	3	48	AKGRGAAATSARL	
			515.8	2	48	GRGAAATSARL	
			371.6	3	43	RLKGLAKKGAA	
			422.7	2	41	PHPHYPP	
			563.8	2	38	SLRPEAASGPAA	
			579.8	2	38	KGRGAAATSARL	
			380.7	2	37	KPPHQGP	
5	0.39	-	477.6	3	47	RDLRTDGKQWR	CNP; *A. feae*; K4IT20

Peak	%	MW (kDa)	Peptide Ion		Score	MS/MS-derived sequence	Protein family/species/accession
			m/z	*z*			
			410.6	3	43	AKGRGAAATSARL	
			424.6	3	39	MVPPKGESVGVR	
			459.6	3	39	AKGRGAAATSARLM	
6	1.49	-	637.8	2	36	DLRTDGKQWR	CNP; *A. feae*; K4IT20
			459.6	3	35	AKGRGAAATSARLM	
			482.7	2	33	GAAATSARLM	
7	0.59	-	509.3	2	32	KPPGVYYPP	CNP; *A. feae*; K4IT20
			425.6	3	32	DLRTDGKQWR	
			460.7	2	26	KPGVYYPP	
8	3.66	-	600.8	2	46	SGPAAAGVGDGWR	CNP; *A. feae*; K4IT20
			491.9	3	42	LAKGRGAAATSARLM	
			557.3	2	42	GPAAAGVGDGWR	
			509.3	2	39	KPPGVYYPP	
9	2.33	-	440.2	3	48	AIVCGGDNPCKK	PLA$_2$; *A. feae*; A0A0D3N8V5
10	0.89	3.8	403.7	2	18	AAAICFR	PLA$_2$; *A. feae*; A0A0D3N8V5
11	35.00	5.0	403.7	2	29	AAAICFR	PLA$_2$; *A. feae*; A7X4P4
			440.2	2	21	AVCECDK	
			552.2	2	30	RQPMDATDR	
12	0.23	28.2	500.3	2	42	IACAPQDVK	SVMP; *Crotalus adamanteus*; ~A0A1W7RB89
			564.3	2	54	KIACAPQDVK	
			376.2	3	31	KIACAPQDVK	
13	0.10	-	494.7	2	46	TDSYPYSR	PLA$_2$; *A. feae*; A0A0D3N9P6
14	1.70	14.3	403.7	2	53	AAAICFR	PLA$_2$; *A. feae*; A7X4P4
			340.5	3	42	TLHYIYGR	
	0.53	12.7	510.8	2	35	TLHYIYGR	PLA$_2$; *A. feae*; A7X4P4
			403.7	2	35	AAAICFR	
			436.7	2	30	NNVIVCR	
			552.2	2	22	RQPMDATDR	
			544.3	2	26	RQPMDATDR	
15	0.69	16.2	1111.6	1	25	NPNPVPTGCR	NGF; *Protobothrops flavoviridis*; ~B1Q3K2
			1293.7	1	77	IDTACVCVISR	
			1809.9	1	74	HWNSYCTTTHTFVR	
	0.58	14.4	340.5	3	42	TLHYIYGR	PLA$_2$; *A. feae*; A7X4P4

Peak	%	MW (kDa)	Peptide Ion m/z	z	Score	MS/MS-derived sequence	Protein family/species/accession
			510.8	2	39	TLHYIYGR	
	0.19	12.6	511.8	2	59	TLHYIYGR	PLA_2; *A. feae*; A7X4P4
			511.9	3	58	CCFVHDCCYGR	
			437.7	2	49	NNVIVCR	
16	0.07	14.9	549.3	2	75	NPNPVPSGCR	NGF; *A. feae*; Q2XXL6
			707.3	2	41	CRNPNPVPSGCR	
17	0.27	14.7	1096.5	1	38	DNLDTYSLR	PLA_2; *A. feae*; A0A0D3N9S4
			1475.6	1	20	CCFVHNCCYR	
18	0.21	15.3	1096.6	1	59	DNLDTYSLR	PLA_2; *A. feae*; A0A0D3N9S4
			1475.6	1	35	CCFVHNCCYR	
			1489.6	1	10	CCFVHNCCYRK	
19	5.89	28.2	1138.5	1	55	SVDFDSESPR	CRISP; *Crotalus horridus*; ~F2Q6E5
			1537.7	1	15	MEWYPEAAANAER	
			1553.7	1	19	MEWYPEAAANAER	
			1905.1	1	121	KPEIQNEIVDLHNSLR	
			2061.2	1	4	KPEIQNEIVDLHNSLRR	
			1138.5	1	21	SVDFDSESPR	
	0.22	19.7	1905.0	1	25	KPEIQNEIVDLHNSLR	CRISP; *C. horridus*; ~F2Q6E7
	2.71	14.4	403.7	2	35	AAAICFR	PLA_2; *A. feae*; A0A0D3N8V5
20	1.49	32.1	1304.6	1	15	NFQMQLGVHSK	SVSP; *A. feae*; A0A1Y0DIB6
			2599.2	1	11	ETYPDVPNCANINILDHAVCQR	
	0.54	14.3	1535.5	1	30	CCFEHDCCYGK	PLA_2; *Bothriechis nigroviridis*; ~C0HJL8
21	2.82	32.2	1304.6	1	22	NFQMQLGVHSK	SVSP; *A. feae*; A0A1Y0DIB6
			2599.2	1	63	ETYPDVPNCANINILDHAVCQR	
			2915.5	1	54	LNSPVHESTHIAPVSLPSSSPTVGSVCR	
	0.07	18.7	2599.3	1	50	ETYPDVPNCANINILDHAVCQR	SVSP; *A. feae*; A0A1Y0DIB6
			2915.6	1	62	LNSPVHESTHIAPVSLPSSSPTVGSVCR	
	0.62	14.5	403.7	2	53	AAAICFR	PLA_2; *A. feae*; A7X4P4
22	0.15	41.6	558.3	2	67	TLCAGILQGGK	SVSP; *Bothrops jararaca*; ~O13069
	0.37	35.4	2599.2	1	31	ETYPDVPNCANINILDHAVCQR	SVSP; *A. feae*; A0A1Y0DIB6
	0.28	32.4	1288.6	1	69	NFQMQLGVHSK	SVSP; *A. feae*; A0A1Y0DIB6
			1304.6	1	34	NFQMQLGVHSK	
			2599.2	1	130	ETYPDVPNCANINILDHAVCQR	

Peak	%	MW (kDa)	Peptide Ion		Score	MS/MS-derived sequence	Protein family/species/accession
			m/z	z			
			2915.5	1	159	LNSPVHESTHIAPVSLPSSSPTVGSVCR	
	0.05	18.4	753.3	2	75	CCFVHDCCYGK	PLA_2; *A. feae*; A0A0D3N8V5
			659.8	2	69	AIVCGGDNPCKK	
			576.9	2	69	EVCECDKAAAICFR	
			595.8	2	66	AIVCGGDNPCK	
			683.8	2	58	MDSYTYSEENK	
			404.7	2	57	AAAICFR	
	0.38	15.0	690.8	2	64	<u>M</u>DSYTYSEENK	PLA_2; *A. feae*; A0A0D3N8V5
			682.8	2	56	MDSYTYSEENK	
			1069.4	2	37	VTGCDPK<u>M</u>DSYTYSEENK	
			752.3	2	48	CCFVHDCCYGK	
			501.5	3	30	CCFVHDCCYGK	
23	0.36	56.0	1513.7	1	200	IIGGDECDINDHR	SVSP; *A. feae*; A0A1Y0DIB5
	0.19	48.6	1117.7	1	31	TLCAGILKGGK	SVSP; *A. feae*; A0A1Y0DIB6
	1.57	40.6	572.3	2	56	EKFFCLSSK	SVSP; *A. feae*; A0A1Y0DIB4
			443.7	2	31	FFCLSSK	
			1116.1	2	17	NSAHIAPLSLPSSPPSVGSVCR	
	0.05	32.7	1095.7	1	14	FLVALYTLR	SVSP; *A. feae*; A0A1Y0DIB4
	0.09	29.6	1003.6	1	20	ALYPGLLEK	SVSP; *A. feae*; A0A1Y0DIB4
			1095.7	1	38	FLVALYTLR	
			2233.2	1	8	NSAHIAPLSLPSSPPSVGSVCR	
			2480.2	1	9	ETYPDVPHCANINIVDHAVCR	
	0.06	28.2	1003.6	1	15	ALYPGLLEK	SVSP; *A. feae*; A0A1Y0DIB4
	0.28	15.9	1003.6	1	12	ALYPGLLEK	SVSP; *A. feae*; A0A1Y0DIB4
			2233.3	1	30	NSAHIAPLSLPSSPPSVGSVCR	
24	12.00	15.6	1505.5	1	10	CCFVHDCCYGK	PLA_2; *A. feae*; A0A0D3N8V5
25	5.56	19.7	998.5	1	29	TDPYPYSR	PLA_2; *A. feae*; A0A0D3N944
			1534.6	1	3	CCFEHNCCYGK	
26	0.07	42.3	414.2	2	17	KVPNEDK	SVSP; *A. feae*; A0A1Y0DIC1
			2929.5	1	99	LNSPVHESTHIAPLSLPSSSPTVGSVCR	
	0.14	28.9	918.5	1	45	VYPGLLEK	SVSP; *A. feae*; A0A1Y0DIC1
			2537.3	1	64	VTHPDVPHCANINILNHAVCQK	
			2929.5	1	119	LNSPVHESTHIAPLSLPSSSPTVGSVCR	

Peak	%	MW (kDa)	Peptide Ion		Score	MS/MS-derived sequence	Protein family/species/accession
			m/z	z			
	0.52	16.9	1022.5	1	36	TLHYIYGR	PLA$_2$; *A. feae*; A7X4P4
			1488.7	1	29	IGLQFYVDAFCR	
			1533.6	1	36	CCFVHDCCYGR	
			1967.8	1	25	NPVTSYSAYGCYCGVGGR	
	0.63	15.6	1022.6	1	46	TLHYIYGR	PLA$_2$; *A. feae*; A7X4P4
			1488.8	1	18	IGLQFYVDAFCR	
			1533.7	1	12	CCFVHDCCYGR	
27	0.43	16.9	1022.5	1	45	TLHYIYGR	PLA$_2$; *A. feae*; A7X4P4
			1488.8	1	50	IGLQFYVDAFCR	
			1533.6	1	42	CCFVHDCCYGR	
	0.68	15.6	1022.6	1	7	TLHYIYGR	PLA$_2$; *A. feae*; A7X4P4
			1488.8	1	3	IGLQFYVDAFCR	
28	3.64	67.9	439.2	2	48	YDTYSTK	LAAO; *Ovophis okinavensis*; ~T2HQ57
			554.3	2	46	VTVTYQTPAK	
			567.3	2	35	YPVKPSEEGK	
29	0.09	156.0	-	-	-	-	Unknown
	1.69	86.0	1059.4	1	53	GQDNFYCR	SVMP; *Sistrurus tergeminus*; ~A0A194ARR8
	0.46	55.9	1388.6	2	1	YSDDYSDDVLDDGMVDHGTKCAVGK	SVMP; *Agkistrodon piscivorus*; ~A0A194ARK7
	0.17	65.9	1852.0	1	29	DSTALFPNIYLETVLR	HA; *A. piscivorus*; ~A0A194ART0
			1889.9	1	6	HSDSNAFLHLFPDSFR	
			2018.0	1	15	KHSDSNAFLHLFPDSFR	
30	0.15	97.3	836.8	2	46	GSQCADGVCCDQCR	SVMP; *C. horridus*; ~A0A0K8RYQ3
			449.2	2	40	TLSHQPSK	
	0.35	60.0	656.3	2	49	SADCPTDDFQR	SVMP; *S. tergeminus*; ~A0A194ARL0
			425.2	2	37	RAGTEC	
31	2.31	-	1458.1	2	1	MARGDNMHDYCNGKTCDCPXNPYK	SVMP; *Echis ocellatus*; ~E9KJX0
32	1.00	54.7	376.9	3	2	VHQMVNIMK	SVMP; *Crotalus atrox*; ~P34182

Methionine oxidation is underlined.
